# Editorial: Can the sharing economy contribute to wellbeing? Exploring the impact of the sharing economy on individual and collective wellbeing

**DOI:** 10.3389/fpsyg.2022.1030430

**Published:** 2022-09-28

**Authors:** Eleni Papaoikonomou, Pia A. Albinsson, Lucie K. Ozanne, Estela Marine-Roig, B. Yasanthi Perera

**Affiliations:** ^1^Faculty of Business and Economics, University of Rovira i Virgili, Tarragona, Spain; ^2^Appalachian State University, Boone, NC, United States; ^3^University of Canterbury, Christchurch, New Zealand; ^4^Universitat de Lleida, Lleida, Spain; ^5^Brock University, St. Catharines, ON, Canada

**Keywords:** editorial, collective wellbeing, individual wellbeing, sharing economy, collaborative consumption

In recent years, the sharing or collaborative economy has received significant attention from scholars and the popular press (Albinsson and Perera, 2018; Huertas et al., 2021). While its potential to create more sustainable, connected and human-centric societies is widely recognized, critics nonetheless note that sharing economy practices engenders negative externalities that must be addressed (Griffiths et al., 2019).

This special issue explores how different facets of the sharing economy can contribute to or hinder individual and societal wellbeing. Wellbeing, a highly researched topic in the field of psychology (Ryan and Deci, 2001), has attracted attention in consumer studies (Lee and Ahn, 2016; Lin et al., 2022), in particular among transformative consumer researchers (Mick et al., 2012; Ekpo et al., 2022). In the extant literature, wellbeing is linked to happiness and life satisfaction (Oral and Thurner, 2019) as well as personal growth, autonomy, and self-acceptance (Ryff and Keyes, 1995). Beyond individual wellbeing, community wellbeing, which focuses on quality of life and group satisfaction, encompasses various dimensions from social and economic to health and environmental wellbeing (McCrea et al., 2016). Emerging research indicates how sharing initiatives can support individual (Albinsson and Yasanthi Perera, 2012; Philip et al., 2019; Ozanne and Ozanne, 2020), family (Ozanne and Ozanne, 2011), and collective or community wellbeing (Albinsson and Yasanthi Perera, 2012; Ozanne and Ozanne, 2016, 2021; Papaoikonomou and Valor, 2016; Ozanne, 2019; Valor and Papaoikonomou, 2019; Albinsson et al., 2021). While being mindful of the broad nature of the sharing economy, the articles presented in this special issue examine how various initiatives within this domain relate to individual and collective wellbeing.

First, in conducting a systematic literature review, Sun and Ertz propose a triple bottom line based (economic, environmental and social aspects) conceptual framework for assessing the initial promises and current sustainability related challenges in the collaborative economy.

Next, we present six empirical articles beginning with three in the context of home sharing. Ozanne and Prayag examine whether hosting through Airbnb, across the private, social and commercial hospitality domains, enhances or diminishes host wellbeing. Their findings indicate that providing hospitality to strangers can both enhance and diminish hosts' wellbeing across material, relational and subjective dimensions. They conclude that various conflicts and tensions arising from providing hospitality diminish hosts' wellbeing, which suggests that the intersection of the private, social and commercial domains generate challenges for these individuals in understanding and managing the host-guest relationship.

Von Richthofen examines how Airbnb hosts' experiences contribute to and detract from their hedonic and eudaimonic wellbeing. He finds that the provision of hospitality, the sociability inherent in host-guest interactions, and guests' positive feedback elicits positive affect in hosts, which contributes to their hedonic wellbeing. However, negative reviews, large numbers of guests, poor guest conduct, as well as dependency on hosting income negatively affects their wellbeing. Moreover, previously unexplored in the sharing economy context, eudaimonic wellbeing is both heightened, and diminished due to being an Airbnb host.

In contrast to Medina-Hernandez et al. utilize online travel reviews to examine value co-creation by users (i.e., guests and hosts) of non-profit sharing accommodation platforms in terms of outcomes, resources, and practices. Their findings indicate that certain tangible and intangible resources, such as the home and its amenities, aid users to co-create value, and that interaction and social practices between guests and hosts creates value for all. Besides indicating that the manner in which each non-profit platform operates affects the nature of value co-creation, this study suggests that relative to for-profit platforms, non-profit accommodation platforms contribute more to the social dimensions of their users' wellbeing.

Next, the focus shifts from home sharing to the issue of how to best promote sharing to enhance happiness and to reduce potential stigma of engaging in such systems. Using an experimental approach, Guo and Lamberton examine whether it is best to frame access-based services for financially-constrained consumers in terms of affordability or variety, which are two popular motives for sharing economy participation. They argue that framing access-based consumption, specifically the renting of clothes, in terms of affordability may undermine financially-constrained individuals' self-image and elicit a sense of poverty stigma thereby decreasing their happiness. Across four studies, the authors provide strong evidence that financially-constrained individuals extract less happiness, reducing their wellbeing, when access-based options are framed in terms of affordability as opposed to variety.

The last research article by Chidimbah et al. focuses on both individual and community wellbeing by examining collective sharing in the form of Village Savings and Loan associations in Malawi. These community programs, which are commonly found in developing countries, are self-managed, self-capitalized and tend to be informal in nature. They allow members to jointly save money and to take small credit loans. As the authors explain, these initiatives are important in providing solutions to major community issues such as deficiencies in the local education system, and the lack of financing for entrepreneurship. In this research, savings emerge as another resource that can be shared at the group or community level to enhance individual quality of life and wellbeing and community resilience.

Lastly, an opinion article by Griffiths et al. focuses on the utilization of collaborative consumption services to address loneliness and social isolation, which are significant public health concerns impacting the health and wellbeing of multiple socio-demographic groups. While sharing opportunities have the potential to develop social ties and enhance community bonds (Ozanne and Ozanne, 2016, 2021) in general, the article discusses initiatives that were specifically developed to address loneliness and social isolation. However, as many sharing economy initiatives entail monetary exchange, the authors highlight potential negative outcomes and call for research on the efficacy and implications of those addressing loneliness and social isolation including the broader societal implications of normalizing companionship for hire.

## Author contributions

All authors listed have made a substantial, direct, and intellectual contribution to the work and approved it for publication.

## Conflict of interest

The authors declare that the research was conducted in the absence of any commercial or financial relationships that could be construed as a potential conflict of interest.

## Publisher's note

All claims expressed in this article are solely those of the authors and do not necessarily represent those of their affiliated organizations, or those of the publisher, the editors and the reviewers. Any product that may be evaluated in this article, or claim that may be made by its manufacturer, is not guaranteed or endorsed by the publisher.
